# Williams Syndrome, Human Self-Domestication, and Language Evolution

**DOI:** 10.3389/fpsyg.2019.00521

**Published:** 2019-03-18

**Authors:** Amy Niego, Antonio Benítez-Burraco

**Affiliations:** ^1^Ph.D. Program, Faculty of Humanities, University of Huelva, Huelva, Spain; ^2^Department of Spanish, Linguistics, and Theory of Literature, Faculty of Philology, University of Seville, Seville, Spain

**Keywords:** self-domestication, Williams syndrome, language evolution, neural crest, language impairment, gene expression

## Abstract

Language evolution resulted from changes in our biology, behavior, and culture. One source of these changes might be human self-domestication. Williams syndrome (WS) is a clinical condition with a clearly defined genetic basis which results in a distinctive behavioral and cognitive profile, including enhanced sociability. In this paper we show evidence that the WS phenotype can be satisfactorily construed as a hyper-domesticated human phenotype, plausibly resulting from the effect of the WS hemideletion on selected candidates for domestication and neural crest (NC) function. Specifically, we show that genes involved in animal domestication and NC development and function are significantly dysregulated in the blood of subjects with WS. We also discuss the consequences of this link between domestication and WS for our current understanding of language evolution.

## Introduction

The evolution of modern language seemingly resulted from multiple changes in the body, the cognitive abilities, and the behavior of our ancestors. No single event can account for such a complex process. Being able to learn and use a language depends on having a brain that is language-ready, that is, which is endowed with specific cognitive abilities that, although rooted in animal cognition, have been improved in our clade as a result of species-specific brain rewiring. At the same time, this ability also depends on living in a particular cultural environment, which has also contributed to the reshaping of the nature of the languages to be acquired and used, as language adapts itself according to the method of transmission (Kirby et al., 2014, 2015, Kirby, 2017). We have good accounts of the genetic and epigenetic changes which occurred after our split from Neanderthals that plausibly account for the emergence of important aspects of our language-readiness (see Boeckx and Benítez-Burraco, 2014a,b and Benítez-Burraco and Boeckx, 2015 for review). We certainly lack confident translations of these changes to the sort of cognitive abilities that are needed for acquiring and mastering a language (but see Murphy and Benítez-Burraco, 2018a,b for some accounts). Regarding the cultural niche that enables (and fosters) language complexity and the acquisition of language by the child, different hypotheses have been launched about its nature and origins. A recent, promising view is that human self-domestication favored the creation of the niche that allowed us to fully exploit the cognitive potential of our language-ready brain, enabling us to learn and accommodate more complex linguistic structures and ultimately, increasing language complexity via a cultural process (Thomas, 2014; Benítez-Burraco et al., 2016; Benítez-Burraco and Kempe, 2018; Thomas and Kirby, 2018).

Since ancient times, it has been observed that humans have much more in common with domesticated animals than with their wild counterparts. Although domestication usually involves selection for tameness, it results in a distinctive set of common features affecting the body and behavior, dubbed ‘the domestication syndrome’ (DS), including floppy ears, shorter muzzles/noses, smaller teeth, smaller jaws, increased docility, earlier sexual maturation and more frequent estrous cycles, reduced sexual dimorphism, neoteny (retention of juvenile characteristics into adulthood), and smaller brains and reduced cranial capacity (Wilkins et al., 2014). These traits are not always present in all domesticated animals, but there are enough incidences of them in enough species to cause us to believe that they are connected to domestication in some way. According to Fitch (2012) and Wilkins et al. (2014), the co-occurrence of these traits results from the hypofunction of the neural crest (NC), which contains stem cells that migrate throughout the body to form the skull and tooth precursors, sympathetic ganglia, adrenal medulla, and other areas of developing vertebrates. Specifically, selection against aggression might cause a sort of “mild neurocristopathy,” inhibiting the proliferation of the neural crest cells (NCCs) at the final sites as a result of changes in cell migration. At the same time, the analysis of the behavioral changes brought about by domestication (but also of their genetic signature) is hindered by the circumstance that many different species, at different moments. As a consequence, some features of domestication are absent in some of them, which leads to difficulties in positing a set of core traits assiciated with domestication. Additionally, the pace of domestication seemingly differs from one species to another, ranging from quick domestication events, like in the silver foxes experiment by Belyaev and colleagues (Belyaev, 1969; Trut, 1999), to the prolonged self-domestication experienced by the human species. This circumstance also complicates the comparisons across species.

All in all, when one looks at anatomically-modern humans (AMHs) in comparison to our primate relatives, but also extinct hominins like Neanderthals, we exhibit reduced cranial robusticity, reduced brain size, reduced tooth size, juvenile cranial shape retained in adulthood, reduced sexual dimorphism, and differences in temperament resulting in less aggressive behaviors (Shea, 1989; Leach, 2003; Somel et al., 2009; Zollikofer and Ponce de León, 2010; Herrmann et al., 2011; Plavcan, 2012; Márquez et al., 2014; Fukase et al., 2015; Stringer, 2016; Thomas and Kirby, 2018), in ways that parallel animal domesticates as compared to their wild conspecifics. Recent genetic research shows that regions under positive selection in AMHs compared to extinct hominins are enriched in candidate genes for domestication in mammals (Theofanopoulou et al., 2017). Morphological signatures of domestication seem to have intensified from 50.000 years ago onward (Bednarik, 2014). Of course, humans were not domesticated in the same sense that other domestic animals were. Instead, we are a self-domesticated species, like some other primate species, particularly bonobos, which also exhibit, compared chimpanzees, many traits of a domesticated phenotype (Cramer, 1977; McHenry, 1984; Shea, 1989; Pilbrow, 2006). Similarly to AMHs, many of the genetic differences found in bonobos compared to chimpanzees concern genes that are related to domestication in other species (Pennisi, 2011). With no external controlling factor triggering the domestication process, it seems that human self -domestication was mostly due to selection against aggression, when humans started to sexually select for non-threatening, less emotionally reactive partners, as a result of the rise of community living, co-parenting and other social factors (Belyaev, 1969; Trut, 1999; Hare et al., 2012; Thomas, 2014; Wilkins et al., 2014). As discussed by Benítez-Burraco and Kempe (2018), the less aggressive behavior associated with self-domestication might have facilitated enhanced intergroup contacts and enhanced learning and teaching behaviors (also favored by the extended juvenile period resulting from self-domestication) that ultimately afforded richer linguistic interactions and ensured mastery (and the creation) of increasingly more complex languages. Eventually, we cannot rule out the possibility that learning more complex language systems had a feedback effect on our cognitive architecture, resulting in the creation of “cognitive gadgets” (Clarke and Heyes, 2017). Importantly, candidates for domestication are related (and partially overlap) with candidates for language readiness (Benítez-Burraco et al., 2016), suggesting that self-domestication might have affected the development and the evolution of our typical brain hardware as well, specifically, our distinctive pattern of brain connectivity and our cognitive abilities, resulting in our language-readiness.

Perhaps not surprisingly, cognitive conditions entailing problems with socialization and language, like schizophrenia (SZ) or autism spectrum disorders (ASD), exhibit an abnormal presentation of traits associated with (self-)domestication; moreover, genes involved in domestication and NC development and function are overrepresented among the candidates for these conditions and/or exhibit altered expression profiles in the brain of affected people (Benítez-Burraco et al., 2016, 2017). This suggests that a deep relationship might exist between cognitive disease, self-domestication, and language evolution. In this paper we explore the links between another cognitive disorder, namely Williams syndrome (WS), human-self-domestication and language (evolution). The physical, behavioral, and cognitive profile of WS shares many important parallels to the DS and contrary to SZ and ASD, its genetic causes are neatly delineated. WS results from a hemizygous deletion of nearly 30 genes on chromosome 7 (Korenberg et al., 2008). Individuals with WS are typically known to be hyper social: they are generally not wary of strangers and are intensely friendly, sometimes overly so (Galaburda et al., 2002). Although WS is thought of as a mental retardation syndrome (Galaburda et al., 2002), people with this condition display an intriguing cognitive profile. Whereas spatial cognition is severely impaired, they excel in musical abilities (Reilly et al., 1990; Udwin and Yule, 1991; Bellugi et al., 1999; Levitin et al., 2005), and outperform subjects with other developmental disorders in language tasks (Karmiloff-Smith and Mills, 2006; Karmiloff-Smith, 2008). Although fine-grained analyses suggest that most components of their language are delayed or impaired (see Karmiloff-Smith and Mills, 2006; Mervis and Becerra, 2007; Martens et al., 2008 for review), their language abilities generally improve with age as a result of compensatory mechanisms, like an increased role of working memory in language processing (Karmiloff-Smith, 1998; Mervis and Becerra, 2007). As we will show in the paper, many of the genes located within the chromosomal region deleted in WS are functionally connected to candidate genes for domestication and NC development and function; in turn, several candidates for domestication and the NC are dysregulated in the blood of subjects with WS. Interestingly, duplications of the WS critical region result in severe speech deficit and oppositional disorder (Morris et al., 2015). Importantly, Korenberg et al. (2000) have identified the WS region as a hotspot in primate evolution, suggesting that it might have played a part in the evolution of human cognition.

Overall, we expect that delving into these links will help us to gain a better understanding of the etiology of WS, but also of the effect of self-domestication in the evolution of our cognitive architecture and the cultural niche that enabled language to evolve, and become more complex. The paper is structured as follows. First, we provide a detailed account of features of domestication in WS, with a focus on physical, cognitive, and behavioral traits. Second, we discuss the role of the genes deleted in WS in NC function, and more generally, in domestication, with a focus on our language-readiness. We examine as well the role of candidates for domestication and NC function found to be dysregulated in the blood of patients with the condition, also with a focus on our mode of cognition and our language abilities. Finally, we discuss the utility (and the limitations) of WS as a model for aspects of language evolution in our species, particularly for the effect of self-domestication. We will conclude that WS (and language deficits in WS) can be viewed as an abnormal ontogenetic itinerary for human cognition (and more specifically, for our faculty of language), resulting in part from changes in genes involved in domestication and NC functioning, and that the etiology of this condition can illuminate aspects of the evolution of language if properly compared with other cognitive disorders.

## Domestic Features in WS

**FIGURE 1:**
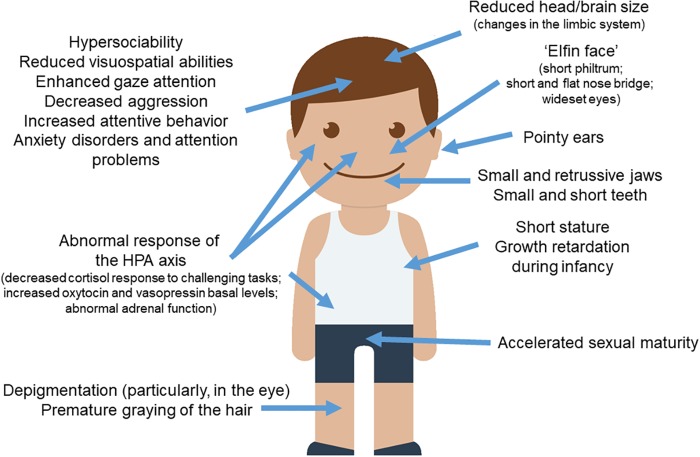
WS and the DS. Most of the clinical features observed in subjects with WS parallels the set of traits found in domesticated strains of animals. The picture of the child was gathered from Iconfinder output (available at http://www.iconfinder.com/icons/525448/boy_child_kid_male_man_person_white_icon).

As noted above, when reviewing the distinctive symptoms of WS, a striking pattern emerges: people with the syndrome seem to exhibit more exaggerated domesticated features than typically developing people, not only in outward physical features, but also related to brain structures and networks, behavior, cognition, and the underlying physiological and biochemical systems that influence them (Figure 1). This gives support for the hypothesis that WS can be seen as a “hyper-domesticated” phenotype in humans. Below we review the parallels between WS and the DS in more detail.

### Physical Anomalies

People with WS exhibit a distinctive “elfin face,” resulting from a short philtrum, pointy ears with longer and narrower conchae, shorter noses with a nose bridge that is flatter than normal, wide mouth, and wideset eyes (Hovis and Butler, 1997; Pober, 2003; Tarjan et al., 2003; Morris, 2010; Poornima et al., 2012). Part of the “elfin” look also results from smaller and retrusive jaws (Axelsson, 2005), which are maintained into adulthood and which resemble the smaller upper jaws observed in domesticated foxes and dogs (Coppinger and Schneider, 1995). As noted by Etchevers et al. (1999) and by Creuzet et al. (2006), jaw size is related to NCCs input. Likewise, numerous studies have observed irregular dentition in WS. In general, the teeth are consistently smaller, shorter, and more widely spaced (Hertzberg et al., 1994; Axelsson, 2005; Moskovitz et al., 2005; Poornima et al., 2012; Maurino et al., 2017). It is also common for individuals with WS to have abnormally small roots, abnormally shaped incisors and molars, and a high number of missing teeth (Tarjan et al., 2003).

### Cognition and the Brain

#### Visuospatial Cognition

One important parallel between WS and DS concerns visuospatial abilities, which are reported to be severely impaired in WS (Bellugi and Wang, 1994; Meyer-Lindenberg et al., 2004; Mobbs et al., 2007; Atkinson and Braddick, 2011; Haas and Reiss, 2012). Usually, people with WS experience problems with seeing the ‘big picture’ made out of smaller components, like a triangle composed of circles (Wang et al., 1995). Interestingly enough, the ability to recognize unfamiliar faces is spared (Bellugi et al., 1992). These deficits have been attributed to the altered size and connectivity of the ventral and dorsal visual processing streams (Meyer-Lindenberg et al., 2004; Mobbs et al., 2007; Atkinson and Braddick, 2011; Key and Dykens, 2011; Haas and Reiss, 2012). Compared to chimpanzees, bonobos also have reduced visual connectivity in certain brain regions, including parts of both the ventral and dorsal visual streams (Rilling et al., 2012). According to Rilling et al. (2012), these differences might also explain why chimpanzees are superior to bonobos in tasks requiring use of tools (van Schaik et al., 1999; Hohmann and Fruth, 2003; Herrmann et al., 2010). Typically, the neurodevelopmental profile of subjects with WS is characterized by difficulties with tool use (see Morris, 2010 for review).

#### Social Cognition

Domesticated animals (dogs vs. wolves, domestic vs. wild foxes, and bonobos vs. chimpanzees) show more attentiveness and sensitivity to human social cues (Hare, 2017). These cues are usually eye or facial movements or gestures. Moreover, they outperform their wild conspecifics only as far as social situations are concerned, but not in general cognition abilities; that is, when cues to problem solving are presented in the form of social interaction (gaze cues, gestures, commands), the more domesticated animals perform better than their wild counterparts, but when the cues are given without social interaction, this advantage disappears (Hare et al., 2002; Hare and Tomasello, 2005; Wobber and Hare, 2009; Lampe et al., 2017). This circumstance lead Hare et al. (2002) to speculate that the process of domestication might create selective pressures for certain social cognition skills only, especially those related to gaze attention, which enable domesticated animals to better communicate with humans. Interestingly, individuals with WS excel in following an experimenter’s gaze to the correct target, although they underperform in the subsequent tasks because they remain fixated on the face and eyes of the experimenter (Riby et al., 2013).

#### Spatial Cognition

Some differences with the neurotypical population can be observed regarding spatial memory, which is more allocentric and less egocentric in people with WS (for instance, they rely more heavily on landmarks when learning a route and retracing it) (Broadbent et al., 2015). According to Broadbent et al. (2015), this could result from the atrophy of certain brain regions, particularly, the hippocampus, a brain structure involved in episodic memory, cognition, spatial navigation, and stress responsiveness (Meyer-Lindenberg et al., 2005a), a phenomenon which is also observed in many domesticates, as reviewed in more depth below.

#### Brain Volume and Brain Regionalization

Individuals with WS have a smaller cranial capacity, and therefore smaller brain volumes compared to controls (Jernigan and Bellugi, 1990; Schmitt et al., 2001a; Reiss et al., 2004; Thompson et al., 2005 Meyer-Lindenberg et al., 2006; Jackowski et al., 2009). This reduction can be broken down into a 15–21% reduction of white matter and a 6–8% reduction of gray matter, which is significant in all lobes (Thompson et al., 2005), with increased cortical thickness in the perisylvian cortex, the superior temporal sulcus, posterior and lateral occipital and inferior occipital temporal regions, and the fusiform area (Thompson et al., 2005). Overall, the WS brain is regionalized differently, and shows altered connectivity almost throughout (Jernigan and Bellugi, 1990), but particularly, regarding white matter pathways associated with social cognition (Haas et al., 2014a). Domestication also entails significant changes in brain structure and function. Domestic animals typically exhibit smaller brains (Kruska, 1988, 2005) that tend to reorganize adaptively in response to the domestic environment. Phylogenetically younger parts of the brain (particularly the forebrain) are more reduced in size under the influence of domestication (Kruska, 1988). Overall, some of the brain areas that seem to be directly related to the cognitive/behavioral phenotype of WS show differences in size, volume and/or structure with the neurotypical population, while others display altered patterns of connectivity. Still others exhibit altered function or altered neurohormone production. In most cases, these are regions that also exhibit structural and/or functional differences between domesticated and wild animals, and/or that are wired differently in both groups. Some parallels of interest for language (in connection to domestication) involve the thalamus, the basal ganglia, parts of the cortex (like the Sylvian fissure) and the limbic system, which we review below.

#### The Thalamus

In people with WS the thalamus is disproportionately reduced (Reiss et al., 2000; Schmitt et al., 2001b; Tomaiuolo et al., 2002; Meyer-Lindenberg et al., 2004). Chiang et al. (2007) and Campbell et al. (2009) found distinct gray matter reduction in the posterior thalamus that might account for their visuospatial deficits, whereas Eckert et al. (2005) suggested that the number of white matter fibers connecting the superior parietal lobule and the posterior thalamus is reduced in subjects with WS. These differences between WS people and the neurotypical population resemble the differences found between chimpanzees and bonobos, given that the latter exhibit a less expansive thalamus, as well as a pronounced decrease of white matter in the medial thalamus (Rilling et al., 2012). As highlighted by Boeckx and Benitez-Burraco (2014a), changes in the thalamus account for crucial aspects of our more globular brain and for our language-readiness. Specifically, Chomsky’s famous ‘Merge’ function, the operation that makes language possible by combining basic linguistic units (Chomsky, 1995), could result from high frequency (e.g., gamma) oscillations being embedded in lower frequency oscillations generated in the thalamus. Significantly, contrary to first reports on this condition, syntax is not spared in WS, but delayed or perhaps impaired across diverse domains (subcategory constraints, passives, Wh-questions, agreement, etc.) (see Karmiloff-Smith and Mills, 2006; Mervis and Becerra, 2007; Karmiloff-Smith, 2008; Martens et al., 2008; for review and discussion).

#### The Striatum

The basal ganglia control many cognitive and emotional functions in humans, including language (Booth et al., 2007; Kotz et al., 2009; Viñas-Guasch and Wu, 2017). Domesticated rats exhibit size reductions of the striatal area (Kruska and Schott, 1977). Likewise, people with WS exhibit decreased volume of the basal ganglia (see Jernigan et al., 1993; Bellugi et al., 1999; Reiss et al., 2000; Campbell et al., 2009), as well as decreased white matter in this area, at least in children (Chiang et al., 2007).

#### The Cortex

The Sylvian fissure has been hypothesized to be partially responsible for the hyper sensitivity that people with WS have to auditory stimuli (Eckert et al., 2006). Many authors (e.g., Kippenhan et al., 2005; Eckert et al., 2006; Gaser et al., 2006; Van Essen et al., 2006; Fan et al., 2017) have reported instances of abnormal gyrification in the Sylvian fissure of subjects with the syndrome. Eckert et al. (2006) found evidence that this atypical Sylvian fissure patterning is associated with the size of the planum temporal, a specific area proximal to the Sylvian fissure which has been related to musicality and perfect pitch in musicians (Hickok et al., 1995). This could explain in part the musical affinity that individuals with WS exhibit. This is a compelling area for research also in terms of their linguistic skills, since Keenan et al. (2001) found that the planum temporal might be a source of linguistic prowess in people with this condition. The depth, position, and shape of the Sylvian fissure has not been explored fully in comparisons between wild and domestic animals, although Schmidt (2014) found that domesticated pigs show a very pronounced, deep Sylvian fissure compared to wild boars. However, this varies greatly in other domesticates such as sheep, horses, or even mink. Likewise, in a study comparing domestic pigeons and their wild counterparts, the rock doves, Rehkämper et al. (2008) found that the nidopallium was smaller in domesticates. The nidopallium, which is highly implicated in both auditory input and vocal output, shares many features with area Spt in humans, located in the Sylvian fissure (Lewandowski et al., 2013).

#### The Limbic System

This is a group of brain structures which support functions like emotion, motivation, and long-term memory (see Rolls, 2015 for review). As noted by Kruska (1988), the most prominent differences between the brains of domesticated animals and their wild conspecifics can be found in the structures comprising this system. The most important components of the limbic system are the hippocampus and the amygdala. Concerning the former, people with WS usually exhibit reduced hippocampal volumes (Meda et al., 2012), as well as an abnormal hippocampal function and response (Meyer-Lindenberg et al., 2006), that might account for their impairment of spatial navigation and especially, of long term spatial and verbal memory. These abnormal features are mimicked in mice models of WS (Segura-Puimedon et al., 2014). Domesticated strains of mammals (laboratory rats, pigs, sheep, poodles, and llamas) show reduced hippocampal volumes as well (and plausibly altered functionality) compared to their wild counterparts (Kruska, 1988, 2005). Regarding the amygdala, evidence has accumulated over the years pointing to this area as the basis for a lot of the emotional abnormalities exhibited by people with WS, in particular, their altered fear processing and their more “friendly” demeanor (i.e., they are less sensitive to fear in social settings, but they generally have more fear and anxiety in non-social situations). Accordingly, the amygdala of subjects with the syndrome underreacts when they have to respond to fearful facial expressions (Meyer-Lindenberg et al., 2005b; Haas et al., 2009), whereas it overreacts when the stimulus is not related to social situations (e.g., spiders) (Jackowski et al., 2009; Muñoz et al., 2010; Capitao et al., 2011). Likewise, the amygdala has a disproportionately larger volume in people with WS in comparison to typically developing people (Reiss et al., 2004; Martens et al., 2009; Järvinen and Bellugi, 2013). Specifically, Haas et al. (2014b) found that individuals with WS have a larger central nucleus of the amygdala, which is a major output area to various other brain areas, including the anterior cingulate cortex, the orbitofrontal cortex, and the prefrontal cortex, which are all important for conscious perception of emotion. Interestingly, as noted by Rilling et al. (2012), compared to chimpanzees, bonobos show a larger amygdala to ventral anterior cingulate cortex (vACC) pathway, which has been found to inhibit aggression by both top-down and bottom-up processes (Davidson et al., 2000; Meyer-Lindenberg et al., 2006; Blair, 2008). Bonobos also show a more enlarged dorsal amygdala (Rilling et al., 2012), a region also implicated in the activation of the hypothalamic–pituitary–adrenal (HPA) axis through connections with the hypothalamus (Davis, 1997; Ledoux, 1998). According to Amunts et al. (2005) and to Barger et al. (2007), this circumstance might explain in part that, similarly to people with WS, bonobos are more anxious when it comes to other, non-social aspects of life, such as eating competition, and have been described as ‘more nervous’ than chimpanzees (de Waal, 1997; Wobber et al., 2010).

The cingulate gyrus is regarded a component of the limbic system, and as such is responsible for certain aspects of emotional response, for regulating aggressive behavior, and for coordinating sensory input with emotions, among many others functions. However, it is also related to language in many ways, especially to language expression, because it contributes to the regulation of the motor functions of speech production via its connections with Broca’s area (Bernal et al., 2015) Compared to chimpanzees, bonobos have a much larger pathway linking the amygdala with the anterior cingulate gyrus (Rilling et al., 2012) and this may account in part for their enhanced empathy and less aggressive impulses. Likewise, they exhibit stronger connections of this region with the amygdala, which seemingly accounts for the increased number of serotonergic neurons found in the bonobo’s amygdala (Stimpson et al., 2016). Haas et al. (2014a) reported that individuals with WS have significantly greater gray matter density in the ventral and dorsal cingulate gyri, among other areas, plausibly because their increased attention to emotional stimuli leads to an increased density of nerve fibers in that area.

The hypothalamus is regarded as a part of the limbic system as well, but its main role concerning the DS results from being an integral part of the HPA axis, as reviewed in the next subsection.

##### Other brain areas of interest

Two other important brain areas deserve to be highlighted because of their connections with the amygdala: the fusiform gyrus and the orbital frontal cortex (OFC). The fusiform gyrus, which is part of the temporal and occipital lobes, is involved in visual recognition. Chimpanzees have a larger right fusiform gyrus compared to bonobos (Rilling et al., 2012). People with WS show smaller total volumes of the fusiform gyrus, although increased volumes of the fusiform face area (FFA), connected to face recognition (Golarai et al., 2010; O’Hearn et al., 2011; Haas and Reiss, 2012). At the same time, their amygdala is less connected to the FFA compared to typically developing subjects (Haas and Reiss, 2012; Vega et al., 2015). Children and adolescents with WS show reduced volumes of gray matter in the left fusiform and increased gray matter volumes in the right fusiform (Campbell et al., 2009) These differences have been claimed to account for their increased focus on face and eyes (Järvinen-Pasley et al., 2008; Haas and Reiss, 2012). In a similar vein, much research on WS points to the OFC and its connection with the amygdala as an important causative factor of the behavioral phenotype of WS (Reiss et al., 2004; Meyer-Lindenberg et al., 2005b; Haas et al., 2009). The OFC is associated with prioritizing behavior in social situations, social cognition, and emotion regulation (Semendeferi et al., 1998; Adolphs, 2003), and is a key zone for the convergence of dorsal and ventral visual stream processing (Suzuki and Amaral, 1994; Epstein et al., 1999). The OFC is involved as well in the processing of empathy (Decety et al., 2008; Carrington and Bailey, 2009; Brink et al., 2011). Some of the most consistent findings from neurobiological research on the WS brain point to differences in the gyral patterns and to reduced gray matter volumes in the OFC (Meyer-Lindenberg et al., 2004; Gaser et al., 2006; Meda et al., 2012; Fan et al., 2017), as well as changes in the white matter pathways connecting this area with the fusiform gyrus and with the amygdala and the hippocampus (Meyer-Lindenberg et al., 2004; Haas et al., 2014a). Compared to less domesticated apes, humans and bonobos have a larger and more diversified posterior OFC (Rilling et al., 2012; Hare and Yamamoto, 2018), which might explain their heightened sensitivity to the mental states of others (see Hare and Yamamoto, 2018 for discussion). Overall, these differences might account of the fact that although subjects with WS are initially very friendly, difficulties with empathy make it difficult for them to sustain social relationships (Plesa Skwerer and Tager-Flusberg, 2016).

### Behavioral Traits and Neuroendocrine Impairment

Typically, domestication entails increased sociopositive behavior, decreased aggression, increased attentive and anxious behaviors, and decreased risk-taking and exploratory behaviors (Kaiser et al., 2015). That said, enhanced sociability is not expressed evenly across the board in all species. On the contrary, it appears in different forms in many domesticated animals: approachability, interest in humans, the ability to read human cues, more elaborate vocalizations, interest in communication with conspecifics, longer fixed gaze patterns, more eye contact with humans, etc. (Deacon, 2009; Wobber and Hare, 2009; Hare, 2017). The social nature of WS encompasses many of these forms linked to the DS. But likewise, this hypersocial profile of people with WS is uneven. Most individuals are eager to interact socially with others, have a higher tolerance of strangers, and an affinity for communicative language (Doyle et al., 2004). Hence, they eagerly strike up conversations or initiate interaction with other people, including strangers. That said, they also experience difficulties with interpreting social cues, sustaining social relationships, and converting empathy into helpful behavior or other types of socially appropriate responses (Plesa Skwerer and Tager-Flusberg, 2016). This might explain the high prevalence of anxiety disorders in people with WS and the reported feelings of isolation despite their attempts to connect with other people (Leyfer et al., 2009; Järvinen and Bellugi, 2013).

The HPA axis is a major neuroendocrine system resulting from complex interactions between the hypothalamus, the pituitary gland, and the adrenal glands, and regulates a great number of bodily functions. In Belyaev’s seminal farm fox experiment, it was shown that the function of the HPA axis was significantly reduced in domesticated foxes in just a few generations, resulting in decreased levels of glucocorticoids, decreased levels of basal adrenocorticotropic hormone in plasma, and reduced adrenal response to stress, which plausibly accounts for the changes in behavior linked to domestication (Naumenko and Belyaev, 1980; Oskina, 1996; Trut et al., 2009). A decreased stress response of the HPA axis was later found in other domesticated animals, like rats and guinea pigs (Kruska, 1988; Künzl and Sachser, 1999; Trut et al., 2009). Specifically, Kaiser et al. (2015) found that although cortisol levels in guinea pigs were similar in wild and domestic strains when it came to basal cortisol activity, the wild strain of cavies exhibited a more pronounced cortisol response when introduced to new environments (Künzl and Sachser, 1999; Künzl et al., 2003; Zipser et al., 2014). The HPA axis also plays a key role in the amygdala response and fear signaling impulses, which also contribute importantly to the behavioral phenotype of domesticated animals. Similarly to domesticated animals, individuals with WS exhibit disrupted HPA axis functions as compared with typically developing subjects. Hence, they show decreased levels of cortisol in situations where evaluation by others is prominent (Lense and Dykens, 2013), but stable cortisol levels during stressful situations that do not depend on others. This effect on the cortisol response is not unexpected if one considers that the output of cortisol involves the amygdala, the prefrontal cortex, and the hippocampus, all of them areas implicated in situations of fear and social stress (see Martens et al., 2008; Dedovic et al., 2009). Occasional adrenal insufficiency has been reported as well in children with WS (Dayal et al., 2017).

The hypothalamus synthesizes two hormones that are of particular relevance regarding domestication (and its parallels with WS): oxytocin (OT) and vasopressin (AVP). These hormones regulate many social behaviors and are involved in most social interactions (Wójciak et al., 2012). Specifically, oxytocin inhibits the HPA axis’ stress triggered activity (Neumann, 2002). Domesticated animals exhibit higher densities of both OT and AVP cells, particularly in the anterior hypothalamus (Ruan and Zhang, 2016). Oxytocin has been related to human-animal interactions, especially when eye contact is involved in communicative settings (see Beetz et al., 2012 for review). According to Nagasawa et al. (2015), dog domestication entailed the borrowing of certain social cognitive traits from humans, particularly, “gaze” behavior, which triggers an ‘oxytocin-mediated positive loop’ that facilitates dog-human bonding. Similarly, in individuals with WS, basal OT and AVP levels are increased, and the increase correlates with social engagement behaviors, such as tendency to approach strangers and emotionality; additionally, OT and AVP release patterns react more markedly in them to positive and negative stimuli (Dai et al., 2012). Higher levels of OT in WS has been hypothesized to result from the hypomethylation (and thus overexpression) of *OXTR*, the gene encoding the oxytocin receptor (Haas and Reiss, 2012), perhaps as a result of the hemideletion of *WBSCR22*, which encodes a methyltransferase (Doll and Grzeschik, 2001; Merla et al., 2002), and/or some effect of *GTF2I*, also deleted in WS, a gene that has been proven to affect the reactivity to OT and ultimately, sociability (Procyshyn et al., 2017). Interestingly, *OXTR* is among the genes that seem to have undergone positive selection in recent hominin evolution (Schaschl et al., 2015). In the neurotypical population higher levels of OT have been shown to increase anxiety (Grillon et al., 2018) or fear of future stress (Guzmán et al., 2013). Not surprisingly in view of its role in social bonding, OT has been related to language evolution, but it happens to be also intertwined with auditory and vocal processing, as well as attention and memory systems (see Theofanopoulou, 2016 and Theofanopoulou et al., 2017 for details). Specifically, OT is involved in social motivation for vocal communication and it might encourage listeners to resolve problems with semantic integration (Ye et al., 2016). Interestingly enough, in children with ASD, higher plasma concentrations of OT correlate with enhanced verbal abilities (Zhang et al., 2016) and the retention of social information, like affective speech (Hollander et al., 2007).

### Other Features

Subjects with WS exhibit some other features typically found in domesticated mammals, like the acceleration of sexual maturation. Pankau et al. (1992) found that individuals with this condition experienced a pubertal growth spurt at age 10 in girls and 13 in boys, which is 1–2 years earlier than the norm (at the time). Pankau and collaborators noted as well that menarche also occurred earlier than normal in girls. Partsch et al. (2002) found similar results in their study of 86 girls with WS, who showed a slightly accelerated sexual maturity. Changes in skin color (specifically, premature graying of the hair) are also found in most subjects with WS, plausibly because of the hemycigosis of *BAZ1B* (Kozel et al., 2014). Likewise, most individuals with the syndrome exhibit less pigmentation in their eyes, as most of them have blue eyes and/or a characteristic “star pattern” in the iris (Jones and Smith, 1975; Greenberg and Lewis, 1988; Holmström et al., 1990).

## Genetic Signatures of Domestication and the Genetics of WS

As noted in the introduction, WS is caused by a hemizygous deletion of 1.5–1.8 Mb on 7q11.23, which affects roughly 30 genes, with >95% patients exhibiting a 1.55 Mb deletion (Pober et al., 2010). In this section we will examine the functional connections between the genes hemideleted in WS and candidates for domestication and NC functioning, as well as the expression pattern of the latter in the blood of subjects with WS, with a focus on aspects of brain function, cognition, and behavior of interest for language (evolution). In order to rely on the most updated list of candidates for domestication, we have merged the list we compiled for our paper on DS in SZ (Benítez-Burraco et al., 2017) with the list delivered by Theofanopoulou et al. (2017). The merged list encompasses 764 genes (Supplementary Table S1). The genes related to NC development and function are the ones also considered on our study on DS in SZ, which comprises 89 genes gathered using pathogenic and functional criteria: neurochristopathy-associated genes annotated in the OMIM database^1^, NC markers, genes that are functionally involved in NC induction and specification, genes involved in NC signaling (within NC-derived structures), and genes involved in cranial NC differentiation (see Supplementary Table S1). Regarding the WS genes, we have considered the 23 protein-coding genes located within the fragment commonly deleted in people with WS, as provided by DECIPHER^2^.

### *In silico* Approach

Among the genes deleted in WS, one finds a robust candidate for domestication in mammals, namely *BAZ1B* (Wilkins et al., 2014). *BAZ1B* plays a key role in chromatin remodeling and nucleosome repositioning (Kitagawa et al., 2003). *BAZ1B* haploinsufficiency dysregulates nearly 50% of the genes expressed in patient-derived neurons (Lalli et al., 2016). *BAZ1B* target gene functions are enriched for neurogenesis and neuron differentiation, and it seems that the gene regulates the balance between neural precursor self-renewal and differentiation (Lalli et al., 2016). Interestingly, *Baz1b* is upregulated in the nucleus accumbens, a key brain reward region, in mice that are resilient to chronic social defeat stress (Sun et al., 2016). Interestingly too, BAZ1B binds the vitamin D receptor (Meng et al., 1998). People with WS suffer from hypercalcemia, which normally resolves after reducing calcium and vitamin D intake (Lameris et al., 2014). Low vitamin D levels correlates with cognitive impairment in clinical conditions involving language deficits, like SZ (Amato et al., 2010) and ASD (Jia et al., 2015). Vitamin D deficiency also reduces the amount of FOXP2-expressing cells in the developing cortex (Hawes et al., 2015); *FOXP2* is a key gene for language development and evolution (Nudel and Newbury, 2013; Graham et al., 2015). Importantly, vitamin D has been hypothesized to play a key role in the emergence of our language-readiness (reviewed in Benítez-Burraco and Boeckx, 2015). Interestingly, it has been claimed that Neanderthals suffered from a vitamin D deficiency (Greenfield, 2015). This deficiency, and more specifically, differences in *FOXP2* regulation by vitamin D, might have contributed, plausibly in subtle ways, to their different social cognition and language abilities (see Benítez-Burraco et al., 2018 for a detailed discussion).

**FIGURE 2:**
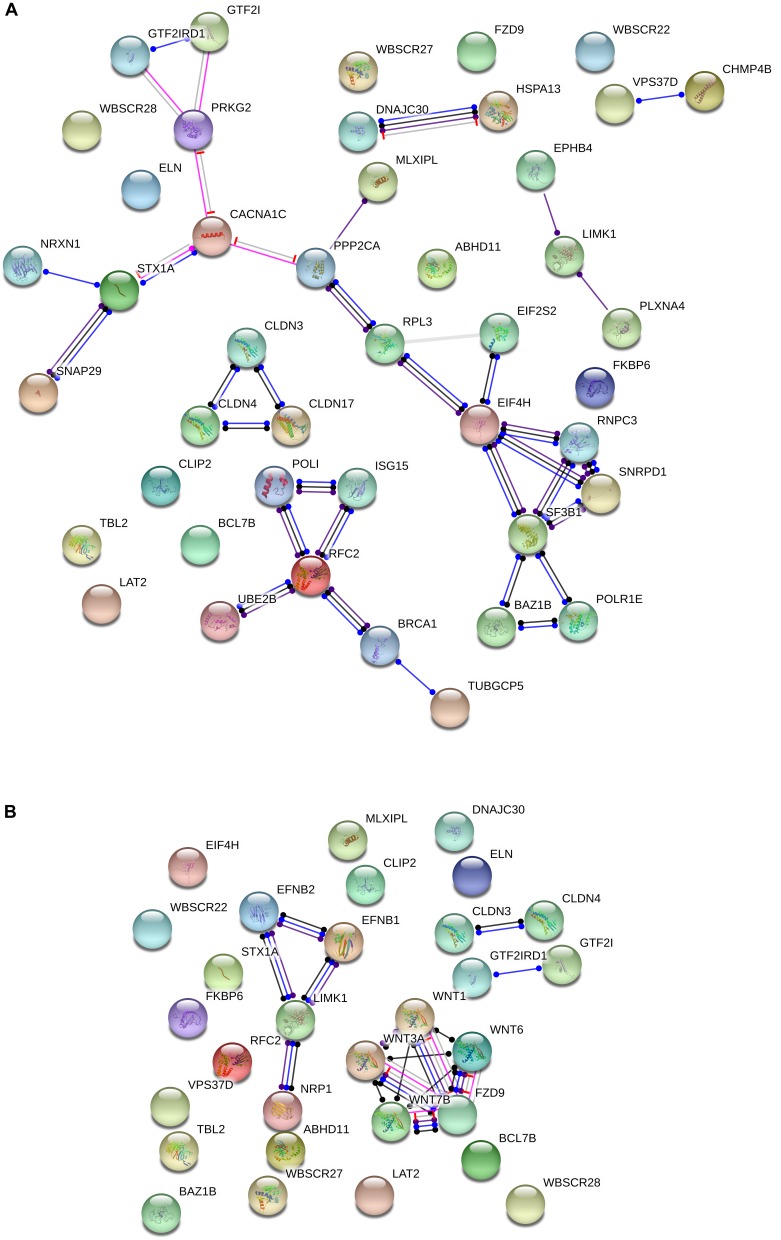
Functional interactions among the WS genes and candidates for domestication **(A)** and NC development and function **(B)**. The diagrams show the network of known functional interactions among the proteins encoded by the genes. The networks were drawn with String (version 10.5; Szklarczyk et al., 2015) license-free software (http://string-db.org/), using the molecular action visualization. Colored nodes symbolize the proteins. The color of the edges represents different kind of known protein-protein associations. Green: activation, red: inhibition, dark blue: binding, light blue: phenotype, dark purple: catalysis, light purple: post-translational modification, black: reaction, yellow: transcriptional regulation. Edges ending in an arrow symbolize positive effects, edges ending in a bar symbolize negative effects, whereas edges ending in a circle symbolize unspecified effects. The medium confidence value was 0.0400 (a 40% probability that a predicted link exists between two enzymes in the same metabolic map in the KEGG database: http://www.genome.jp/kegg/pathway.html). The diagram only represents the attested connectivity between the involved proteins, derived from curated databases or experimentally determined, but it has to be mapped onto particular biochemical networks, signaling pathways, cellular properties, aspects of neuronal function, or cell-types of interest to gain a more accurate view of its relevance for the presentation of domesticated features in WS (see the main text for details).

Additionally, several of the genes within the WS critical region interact with candidates for domestication in mammals. Specifically, String 10.5^3^ points to attested functional interactions between nearly one third of the genes deleted in WS and more than 20 candidates for domestication (Figure 2A). The most interesting set of connections are the links between the WS genes *BAZ1B, EIF4H, GTF2I, GTF2IRD1, MLXIPL*, and *STX1A*, and the domestication candidates *PRKG2, CACNA1C, NRXN1, SNAP29, PPP2CA, RPL3, EIF2S2, RNPC3, SNRPD1, SF3B1*, and *POLR1E*. Specifically, *GTF2I* has been related to cognitive problems and craniofacial abnormalities in WS (Morris et al., 2003; Tassabehji et al., 2005). One of its functional partners is *USF1* (Roy et al., 1997), whose regulatory region has undergone 30 fixed or high frequency changes after our split from Denisovans (Meyer et al., 2012). As noted above, *GTF2I* has been found to affect sociability and anxiety through modulating the oxytocin reactivity (Dai et al., 2009; Brunberg et al., 2013; Procyshyn et al., 2017). Mice with a heterozygous deletion of *Gtf2i* tend to have a greater interest in social interactions with unfamiliar mice, but reduced interest in new objects, mirroring what is observed in subjects with WS (Sakurai et al., 2011). The involvement of this gene in brain function and cognition seemingly results from the fact that Gtf2i is an upstream regulator of various brain processes, including neuronal development, inhibitory synapse maturation, and neural circuit formation (Shirai et al., 2017). Likewise, a heterozygous deletion of *Gtf2ird1*, which encodes a repressor of *GTF2I* transcriptional functions, results in hypersociability, as well as in learning, and memory deficits in mice (Tassabehji et al., 2005; Young et al., 2008; Palmer et al., 2010). The hemyzigosis of *GTF2IRD1* and *GTF2I* has been hypothesized to contribute as well to the cognitive and language features of WS (Vandeweyer et al., 2012), because it gives rise to motor dysfunctions and vocalization alterations (Howard et al., 2012). Regarding the candidates for domestication belonging to this network, it is worth highlighting that *PRKG2* has been associated with dwarfism in livestock (Boegheim et al., 2017), but also to spatial memory and motor coordination deficits in mice (Wincott et al., 2013), and to intellectual disability and speech problems in humans (Bonnet et al., 2010; Hu et al., 2017). Signaling through *Prkg2* in the amygdala is critical for auditory-cued fear memory and long-term potentiation (Paul et al., 2008). *CACNA1C* encodes the alpha 1C subunit of the Cav1.2 voltage-dependent L-type calcium channel (Kumar et al., 2015) and has been related to deficient semantic verbal fluency in SZ (Krug et al., 2010), as well as to executive dysfunction, intellectual disability, and ASD (Damaj et al., 2015). The hypermethylation of *CACNA1C* in AMHs compared to Neanderthals is suggestive of increased cross-frequency coupling between specific brain oscillations (θ and γ bands), and ultimately, of enhanced working memory operations in our species across a number of modalities (see Murphy and Benítez-Burraco, 2018a for details). *NRXN1* encodes a neurexin that regulates synaptic activity, neuritogenesis, and neuronal network assembly during neocortical development (Süudhof, 2008; Gjørlund et al., 2012; Jenkins et al., 2015). Mutations in the gene are known to impair speech severely, and give rise to mild motor delay too (Zweier, 2012). Mutations in *SNAP29* causes cerebral dysgenesis (Sprecher et al., 2005). The gene contributes to the modulation of synaptic transmission (Su et al., 2001; Pan et al., 2005). *PPP2CA* is involved in the regulation of axonal growth (Li et al., 2018). *SF3B1* is a candidate for SZ (Schizophrenia, 2014). The gene is associated with *PQBP1*, linked to developmental delay and microcephaly (Li et al., 2013) and to intellectual disability (Wang M.J. et al., 2013).

Another interesting network is comprised by the WS gene *RFC2* and the candidates for domestication *BRCA1*, *ISG15*, *POLI*, *UBE2B*, and *TUBGCP5*. *BRCA1* is highly expressed in the embryonic neuroepithelium when neural progenitors are highly proliferative, playing distinct apoptotic and centrosomal functions important for the developmental regulation of brain size (Pao et al., 2014). *ISG15* is a FOXP2 target (Vernes et al., 2011) and encodes an error-prone DNA polymerase involved in DNA repair. *TUBGCP5* has been related to Attention Deficit-Hyperactivity Disorder (ADHD) and Obsessive-Compulsive Disorder (OCD; Picinelli et al., 2016), as well as to ASD (Sanders et al., 2012).

Regarding the NC, no genes within the WS region are listed among the candidates for NC development and function. Nonetheless, *BAZ1B* is hypothesized to upregulate some of the genes required for the embryonic growth of NCCs in humans, like *SNAIL* and *SLUG* (Barnett et al., 2012). Knockdown of *BAZ1B* also results in *SOX2* downregulation (Corley et al., 2016). *SOX2* is a robust candidate for domestication (Wilkins et al., 2014). Also, it is functional in various NCCs, having an instrumental effect on skin regeneration (Johnston et al., 2013) and sensory neurogenesis (Cimadamore et al., 2011). Likewise, both *GTF2I* and *GTF2IRD1* are expressed in NC-derived tissues, with heterozygous deletions of either gene giving rise in mice to craniofacial defects (Enkhmandakh et al., 2009). Significantly, *GTF2I* is also involved in ASD, through DLX5/DLX6 regulation (Shirai et al. (2017), which are two genes important for the changes resulting in our language-readiness (Boeckx and Benítez-Burraco, 2014b).

Moreover, although several other connections can be found in the literature, we wish to highlight the functional links pointed out by String between some genes within the WS region and candidates for NC, particularly, between *LIMK1* and *EFNB1*, *EFNB2*, and *NRP1*, and between *FZD9* and *WNT7B*, *WNT3A*, *WNT1*, and *WNT6* (Figure 2B). *LIMK1* encodes a serine/threonine kinase involved in many cellular processes associated with cytoskeletal structure, including axon growth and brain development and function. Specifically, *LIMK1* has been shown to regulate long-term memory and synaptic plasticity (Todorovski et al., 2015). In combination with some other of the genes within the WS fragment, *LIMK1* has been related to the visuospatial problems experienced by people with WS (Gray et al., 2006; Smith et al., 2009), but also with the approachability which is typical of their social cognition (Hoeft et al., 2014). *EFNB1* is among the most common single gene causing syndromic craniosynostosis and other clinical conditions involving abnormal skull/face development, specifically, craniofrontonasal syndrome (Wieacker and Wieland, 2005; Kimonis et al., 2007). *EFNB2* encodes a component of the Reelin pathway, important for brain development, and is also a target of *PAX6* in the forebrain (Xie et al., 2013). *PAX6* contributes to the regulation of the migration of NCCs from the anterior midbrain (Matsuo et al., 1993) and is a FOXP2 target (Konopka et al., 2011). Finally, *NRP1* encodes a neuropilin that regulates many aspects of neural development, including neuronal migration, axon patterning, and synaptogenesis; specifically, it helps to guide the NCCs precursors of neurons and glia in the peripheral nervous system (Raimondi and Ruhrberg, 2013). Regarding the WS gene *FZD9*, it is critical for hippocampal development (Zhao et al., 2005), but also contributes to regulating cell division and programmed cell death (Chailangkarn et al., 2016). *FZD9* is a receptor for several components of the WNT family and this interaction is important for the involvement of NC in body development, particularly, in the early development of the central nervous system (Wang et al., 2010; Ossipova and Sokol, 2011). The hemizygosis of *FZD9* has been hypothesized to result in longer dendrites, increased numbers of spines and synapses, aberrant calcium oscillation, altered network connectivity, and enhanced glutamatergic excitatory synapses (Chailangkarn et al., 2016). FZD9 direct ligands WNT7B, WNT3A, WNT1, and WNT6 are different members of the WNT family with important roles in brain development. Specifically, *WNT7B* controls neuronal differentiation and the development of forebrain structures by regulating the expression of selected pro-neural transcription factors (Papachristou et al., 2014). *WNT3A* exerts a neuroprotective effect in several brain areas, including the hippocampus (Zhao et al., 2016; Ríos et al., 2018). Both *WNT7B* and *WNT3A* are involved in the pre-synaptic assembly (Ahmad-Annuar et al., 2006; Cerpa et al., 2008; Davis et al., 2008). *WNT1* plays a role in the induction of the mesencephalon and cerebellum and has been related to fear memory formation and long-term memory consolidation in the amygdala (Maguschak and Ressler, 2011). Finally, *WNT6* is involved in craniofacial morphogenesis (Hu and Marcucio, 2009).

Overall, the evidence reviewed in this section supports the view that the hemideletion of the WS fragment can result in a significant alteration of many genes related to DS and NC, providing a genetic rationale of the parallels between the DS phenotype and the WS clinical profile.

### *In vivo* Approach

The data discussed in the previous subsection suggest that some genes either interact with or are themselves, candidates for domestication in mammals and/or NC development and function (Supplementary Table S1) and that this circumstance might account for the abnormal presentation of features of domestication in people with this condition. Still, these connections were found in the literature and/or were uncovered *in silico*, based on curated databases of interactions between proteins and on experimentally determined interactions. In order to establish the biological reliability of these links, and ultimately, of our hypothesis, we conducted a more physiologically focused analysis, relying on gene expression profiles in the blood of people with WS. Our aim was to know whether genes involved in domestication and NC development and function are dysregulated in this condition and whether this up- or downregulation can explain aspects of the WS phenotype, particularly, their abnormal features of self-domestication. The gene expression profiling data of peripheral blood in patients with WS was obtained from Gene Expression Omnibus (GSE 89594). We then used the Benjamini-Hochberg method (Benjamini and Hochberg, 1995) to calculate the false discovery rate (FDR). Genes were considered to be differentially expressed genes (DEG) when the FDR < 0.05 and the | fold change (FC)| > 1.2. We evaluated the statistical overrepresentation using Fisher’s exact test.

**FIGURE 3:**
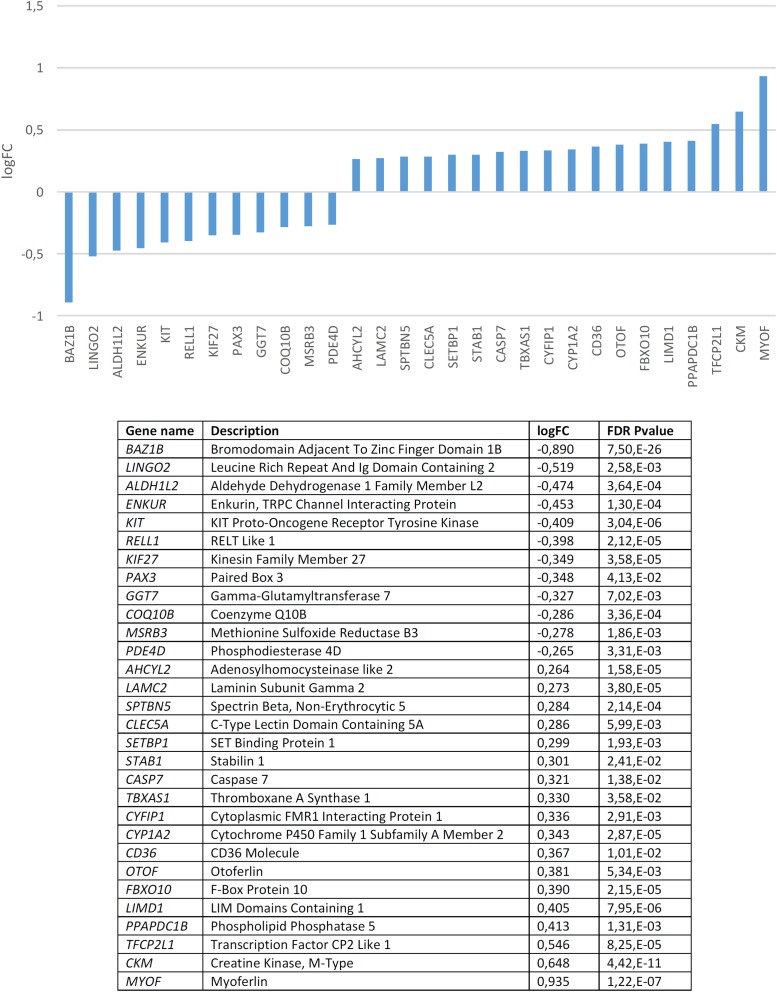
Candidates for domestication that are significantly dysregulated in the blood of subjects with WS (FDR < 0.05, | FC| > 1.2).

We found that candidates for domestication are not significantly dysregulated in the blood of subjects with WS (*p* = 0.20 by Fisher’s exact test). Nonetheless, several genes are significantly up- or down-regulated compared to controls (Figure 3). In order to check the specificity of this set of genes in relation to domestication and to features of the WS phenotype, we conducted a functional enrichment analysis with Enrichr^4^ (Chen et al., 2013; Kuleshov et al., 2016). Our results (Supplementary Table S2) show that the dysregulated genes mainly contribute to bone development, vitamin and hormone homeostasis, lipid metabolism, and skin development, which are all aspects found affected in people with WS. Regarding their molecular function, they typically participate in low-density lipoprotein activity, but also in protein modification (via phosphodiesterase modification), gene regulation (via histone modification), and cytoskeleton assemblage (via dynactin binding). Perhaps not surprisingly, in mammals the alteration of these genes result in phenotypes that mimic aspects of the DS, particularly changes in pigmentation and body size. Interestingly too, they are associated in humans to clinical symptoms mostly related to hair and eye pigmentation, craniofacial and limb morphology (like malar flattening, mandibular prognathia, and finger camptodactyly), and hormone homeostasis (hypothyroidism). Finally, these genes are predicted to be preferentially expressed in the gut, but also in the blood and the brain. According to the Human Brain Transcriptome Database^5^ all these genes are expressed in the brain, particularly in the cerebellum (see Supplementary Table S3). The cerebellum is crucially involved in language processing (Vias and Dick, 2017; Mariën and Borgatti, 2018) and subjects with WS exhibit cerebellar volume alterations that are associated with their cognitive, affective and motor distinctive features (Osório et al., 2014).

Among the candidates for domestication found downregulated in the blood of subjects with WS, besides *BAZ1B* it is worth highlighting several other genes. *KIT* is one of Wilkins et al. (2014) candidates for domestication. This gene encodes a tyrosine kinase receptor which plays a key role in the regulation of NC-derived processes, like melanogenesis or hematopoiesis (Rothschild et al., 2003; Kasamatsu et al., 2008). In rats, mutations in *Kit* impair hippocampal synaptic potentiation and spatial learning and memory (Katafuchi et al., 2000). *KIF27* encodes a putative ciliary motor with an important role in the primary cilia, which interacts with Gli transcription factors (Wilson et al., 2009; see Cohen, 2010 for discussion). GLI factors have been hypothesized to play a key role in events resulting in our skull/brain globularization, self-domestication, and language-readiness (Boeckx et al., unpublished). *PAX3* is another of Wilkins et al. (2014) candidates for the DS in mammals and interacts with two other core candidates, namely *SOX10* (Lang and Epstein, 2003), and *TCOF1* (Barlow et al., 2013). *PAX3* encodes a transcription factor involved in neural development, myogenesis, and craniofacial patterning, and it is among the earliest genes activated in NC progenitors (Maczkowiak et al., 2010; Bae et al., 2014; Plouhinec et al., 2014). This gene is a candidate for Waardenburg syndrome, a clinical condition entailing sensorineural hearing loss and developmental delay (Tassabehji et al., 1992; Chen et al., 2010). It has been associated as well with orofacial cleft in distinct populations (Böhmer et al., 2013; Butali et al., 2014; Leslie et al., 2015, 2016; Gowans et al., 2016). *MSRB3*, which encodes a methionine sulfoxide reductase, has been shown to contribute to the regulation of hippocampal volumes in selected regions along the dentate gyrus, subiculum, CA1 and fissure (Hibar et al., 2017). Specifically, the MSRB3 protein is found to be associated with synaptic vesicles, particularly, in the neuropil of the CA1 pyramidal layer (Adams et al., 2017). Interestingly, in patients with Alzheimer’s disease, the MSRB3 protein is more abundant in the soma of the neurons (Adams et al., 2017). Additionally, *MSRB3* deficiency causes hearing loss due to stereocilia degeneration and apoptotic death of the cochlear hair cells (Kwon et al., 2014; Kim et al., 2016). *PDE4D* encodes a phosphodiesterase that degrades cAMP, contributing to the regulation of its physiological role in specific brain pathways and in different brain areas, including the hippocampus and the basal ganglia (Miró et al., 2002). In particular, PDE4D modulates at synapses the role of DISC1, a protein related to SZ (Bradshaw et al., 2008). In the mouse brain, *Pde4d* is highly expressed in the cerebellum and the thalamus (Cherry and Davis, 1999). Inhibition of *Pde4d* enhances neuronal plasticity and memory (Ricciarelli et al., 2017; Zhang et al., 2018).

Among the candidates for domestication found to be upregulated it is worth highlighting *SETBP1*, which encodes a SET binding protein and which is a candidate for specific language impairment (SLI). GWAs have associated this gene with the complexity of linguistic output (Kornilov et al., 2016). Microdeletions affecting *SETBP1* have been shown to impact mostly on expressive abilities, whereas receptive abilities remain substantially preserved, to the extent that some patients can communicate through miming and gestures (Filges et al., 2011; Marseglia et al., 2012). Common polymorphisms in *SETBP1* have been recently associated with reading abilities in the neurotypical population, particularly, with phonological working memory, via the activation of the right inferior parietal lobule (Perdue et al., 2018). Mutations on the gene result as well in social and behavioral problems (Coe et al., 2014). This gene is also a candidate for Schinzel-Giedion syndrome, a condition entailing severe developmental delay and occasional epilepsy (Ko et al., 2013; Miyake et al., 2015). Likewise, *STAB1*, which encodes a scavenger receptor for acetylated low density lipoproteins, with an important role in defending against bacterial infections, has been found to be associated with conditions impacting on our distinctive cognitive abilities, including bipolar disorder (Witt et al., 2014), Alzheimer’s disease (Giil et al., 2017), and pediatric Multiple Sclerosis patients (Liguori et al., 2019). *CASP7* encodes a caspase that contributes to the cleavage of nuclear substrates during neuronal apoptosis (Hayashi et al., 2006). The gene has also been shown to influence early neurodegenerative changes, particularly, as observed in Alzheimer’s disease (Sloan et al., 2010). *TBXAS1* encodes an endoplasmic reticulum membrane protein that catalyzes the conversion of prostaglandin H2 to thromboxane A2, a potent vasoconstrictor, and it has been associated with gray matter volume differences in the cortex and the cerebellum of schizophrenic patients (Wang Q. et al., 2013). *CYFIP1* regulates presynaptic activity during development, as well as electrical activity in the hippocampus (Hsiao et al., 2016). Increased *CYFIP1* dosage alters cellular and dendritic morphology (Oguro-Ando et al., 2015) and the gene is thought to play a critical role in the maintenance of dendritic complexity and the stabilization of mature spines (Pathania et al., 2014). *CYFIP1* is a candidate for several clinical conditions impacting cognitive and social abilities, including epilepsy, SZ, intellectual disability, and ASD. Hence, reduced *CYFIP1* levels in neural progenitors result in dysregulation of SZ and epilepsy gene networks (Nebel et al., 2016). Likewise, the gene is upregulated in intractable temporal lobe epilepsy patients (Huang, 2016), and also in the blood of people with ASD (Noroozi et al., 2018). In mice, *Cyfip1* haploinsufficiency results in decreased dendritic spine density and stability, and altered synaptic plasticity, as well as in motor learning deficits (Bachmann et al., 2019). Additionally, CYFIP1 promotes in the brain the translation repression activity of FMR1, the main causative factor of X-Fragile syndrome, to the extent that haploinsufficiency of *Cyfip1* produces fragile X-like phenotypes in mice (Bozdagi et al., 2012). Interestingly, a common variant of *CYFIP1* has been associated with structural variation at the language-related left supramarginal gyrus (Woo et al., 2016). *CYP1A2*, which encodes a member of the cytochrome P450 superfamily, has been associated with super-refractory SZ (de Brito et al., 2015). *CD36* encodes a fatty acid translocase with a key role in the transport and intracellular trafficking of fatty acids and in energy homeostasis in the brain, but also in cognitive processes, like learning abilities (see Moullé et al., 2012 for review). Lastly, *OTOF* encodes a calcium ion sensor involved in the control of neurotransmitter release at ribbon synapses of cochlear hair cells (Pangršič et al., 2012). Mutations in *OTOF* cause neurosensory nonsyndromic recessive deafness (Santarelli et al., 2015).

**FIGURE 4:**
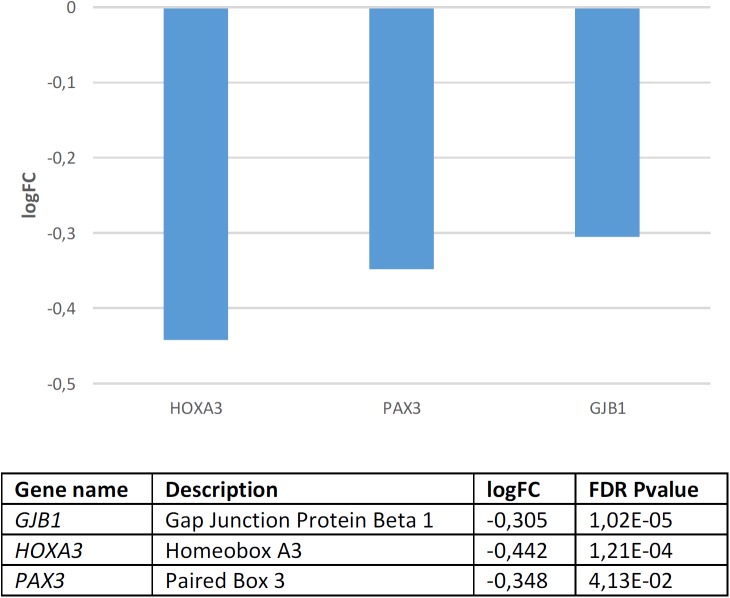
Candidates for NC development and function that are significantly dysregulated in the blood of subjects with WS (FDR < 0.05, | FC| > 1.2).

Regarding candidates for NC development and function, we found that they are significantly dysregulated in the blood of subjects with WS (*p* = 4.0e-3 by Fisher’s exact test). Several genes are significantly downregulated compared to controls (Figure 4). According to Enrichr (Supplementary Table S4), these genes contribute significantly to cell assembly, sensory perception, gene expression, and nervous system development. Concerning their molecular function, they regulate channel activity and DNA binding. In mammals, mutations in these genes result in an abnormal development of different body organs, including the thyroid gland, the tongue, the spinal column, the ear, the larynx, the thymus, and the heart, but also in pigmentation anomalies. In humans, they are mostly associated with pigmentation changes in the hair, the eye, and the lips, as well as to ear and lung dysfunction, and altered craniofacial and skeleton morphology. Finally, although these are NC genes, they are also predicted to be expressed in the branchial arches, the muscles, and other body organs, like the pharynx and the thymus. According to the Human Brain Transcriptome Database, all the NC genes that are downregulated in the blood of patients with WS are expressed in the brain (Supplementary Table S5).

*GJB1* which encodes a member of the gap junction protein family, involved in the transference of ions and small molecules between cells, is the main candidate for X-linked Charcot-Marie-Tooth disease type 1, a type of hereditary motor and sensory neuropathy (Kleopa et al., 2012). Some mutations in the gene have been reported to cause transient central nervous system dysfunction, including dysarthric speech (Siskind et al., 2009). *HOXA3*, which encodes a transcription factor involved in gene expression regulation, morphogenesis, and differentiation, regulates the migration of branchial nerve precursors (Watari-Goshima and Chisaka, 2011). Finally, *PAX3* is a core candidate for domestication discussed above.

Overall, these findings provide experimental support for the hypothesis that the WS phenotype results in part from the dysregulation of selected candidates for domestication in mammals and for NC development and function. Certainly, because our focus of interest is put on language (see section “WS, Domestication, and Language Evolution”), most of the relevant changes are expected to concern to the brain, but not to the blood. Nonetheless, a significant overlap between both tissues exists, ranging from 20% (Sullivan et al., 2006; Rollins et al., 2010) to 55% (Witt et al., 2013). Accordingly, we regard that our findings in the blood can be confidently extrapolated to the brain.

## WS, Domestication, and Language Evolution

As noted in the introduction, signs of domestication can be found in AMHs compared to extinct hominins and these signs have seemingly been exacerbated in the last 50.000 years, in a period when important changes in our behavior occurred. Although the regions with signals of positive selection in our species are enriched in candidates for domestication (Theofanopoulou et al., 2017), we still lack a good understanding of the effect of these changes in our cognitive and behavioral phenotype. First, the timing of these selective sweeps is not clear. Did they occur in late AMHs, thus potentially accounting for the enhanced self-domestication of the human species in our recent history and ultimately, for all the expected changes in our cognition and language abilities? Or did they occur in early AMHs, therefore having a more indirect effect on our cognitive architecture and behavior? Second, we have no idea about the biological consequences of these DNA changes. Did they result in real changes in the expression levels of the affected genes? And if they did, did they have a measurable impact on body tissues involved in the DS? On which one(s)? Specifically, should we expect a direct effect of these changes on brain function, or should we instead expect that the cognitive changes linked to self-domestication had a more indirect source, perhaps resulting from behavioral changes? Fortunately, we are not totally in the dark. As far as language is concerned, candidates for domestication have been shown to be intimately linked with the set of genes accounting for our language-readiness, which seemingly changed after our split from Neanderthals (Benítez-Burraco et al., 2017; see Mozzi et al., 2016 for a different account of these changes). Accordingly, one could expect that the changes that habilitated the neuronal workspace enabling our cognitive modernity, including our ability to learn and use languages, also contributed to the changes resulting in our species-specific cultural niche linked to human self-domestication, which contributed in turn to increase language complexity via a cultural change. The inverse is also true, of course, because our mode of cognition, mostly resulting from biological changes, has a significant impact on our cultural practices. Importantly too, cultural changes can also affect our cognitive architecture via the creation of “cognitive gadgets” through subtle modifications in learning and data-acquisition mechanisms like attentional focus or memory resources (Clarke and Heyes, 2017; Lotem et al., 2017). That said, it is clear that we still need to disentangle the complex relationships that seemingly exist between genes (in particular, candidates for language-readiness and candidates for domestication), behavior, and the environment (physical and cultural), which we expect account for the evolution of language (and languages).

Certainly, an increasing body of research deals with the consequences of domestication for language evolution (see Benítez-Burraco and Kempe, 2018, Thomas and Kirby, 2018 among many others). Nonetheless, it necessarily builds on indirect evidence and results in hypotheses that are difficult to prove. Language disorders, if analyzed at the proper level of granularity (genes, protein networks, and the like) can serve as a confident window onto language evolution, because of the robust link between developmental disturbances and evolutionary history (see Benítez-Burraco and Boeckx, 2014 for discussion). Interestingly, altered features of domestication have been shown to be related (and perhaps contribute to) several cognitive disorders entailing problems with language, like ASD and SZ (Benítez-Burraco et al., 2016, 2017). It is our contention here that WS could be the best available model when it comes to use cognitive disorders for testing current hypotheses about the effect of domestication in language evolution. First, as we show in the paper, it simultaneously entails cognitive and language alterations, and increased features of domestication. Second, it might help disentangle the effect of specific processes associated with domestication. As discussed by vonHoldt et al. (2017), domestication does not entail an increased ability for social problem solving as such. Actually, human-socialized wolves can outscore domestic dogs across many sociocognitive domains (Udell et al., 2010). It is their enhanced hypersociability, the main distinctive feature of people with WS, that distinguishes dogs from wolves (Udell et al., 2010). Third, studies looking for genomic signals of domestication, which compare wild and domesticated variants of mammals, regularly find genes related to WS among the ones that have changed in domesticated animals. The whole region ortholog to the WS region is under positive selection in domestic dog breeds (vonHoldt et al., 2010), and recent studies highlight *GTF2I* and *GTF2IRD1* as the genes that might explain the enhanced sociability of dogs compared to wolves (vonHoldt et al., 2017). Likewise, comparisons between wild and domesticated foxes have found positive selection of three genes located at the border of the WS deletion in tamed foxes (Kukekova et al., 2018). And as we have shown in the previous section, WS genes are functionally connected to many candidates for domestication and NC function. Moreover, some of these candidates are dysregulated in the blood of subjects with WS. The “neural crest hypothesis” of domestication (Wilkins et al., 2014) predicts a reduced expression of genes affecting NC development in domesticated animals. Actually, this is what we have found in the blood of patients with WS. Interestingly, there exists a mirror condition to WS, the so-called 7q11.23 Duplication syndrome, resulting from the duplication of the region deleted in WS. As noted by Morris et al. (2015), subjects with this condition exhibit opposite features to people with WS, including macrocephaly, speech problems, and impaired social cognition. This circumstance suggests that gene dosage is a key factor accounting for the differences between these two phenotypes, and more generally, that the hypothesis of self-domestication as a result of the hypofunction of the NC (and the downregulation of selected genes) might be on the right track. Fourth, the WS region seems to be a hotspot for genomic evolution in primates, with many species-specific duplications and rearrangements that resulted in significant differences among primate genomes (Antonell et al., 2005). It has been hypothesized that transposon activity (Alu-mediated duplicated transposition) might account for these changes and diversity (Antonell et al., 2005). Interestingly, in canines, transposon dynamics have been associated with a hypersocial behavioral syndrome and among the transposon-derived sequences that are hyper-methylated in dogs compared to wolves (and potentially downregulated in them), one finds several of the WS genes (*WBSCR17*, *LIMK1*, *GTF2I*, *WBSCR27*, *BAZ1B*, and *BCL7B*) (vonHoldt et al., 2018). Finally, WS has a well-defined etiology, in contrast to other cognitive disorders entailing abnormal cognitive and linguistic features and abnormal domesticated features, like ASD or SZ, for which hundreds of candidate genes have been posited. That said, although all these circumstances seemingly corroborate the utility of the study of the WS region for understanding how humans became self-domesticated (and how language evolved), some caution is in order. Not every single neuropsychiatric condition can be fully explained in the framework of the domestication hypothesis. After all, disorders are adaptive response to specific gene alterations that seemingly affect the whole brain (and the body) (Boyle et al., 2017). Also, not every human physical and cognitive trait can be linked to our self-domestication or be interpreted as an adaptive response to the conditions that triggered our self-domestication, because other factors shaped our evolutionary history as well.

In this final section of the paper we will discuss parallels between aspects of WS behavior and language, and behaviors that we have highlighted as foundations of cultural transmission processes that may have facilitated the emergence of modern languages, specifically, parenting and teaching behaviors, and play behavior (see Benítez-Burraco and Kempe, 2018; Langley et al., unpublished). Certainly, people with WS exhibit mental retardation and cognitive deficits that have a negative impact on their language. At the same time, however, their language exhibits some of the features we have hypothesized as resulting from cultural learning and more generally, from human self-domestication. The fact that there is an overlap between genetic signatures of language evolution, domestication, and WS also reinforces this view.

Increased socialization resulting from human self-domestication has been hypothesized to push language change toward language systems optimized for conveying decontextualized information between unfamiliar individuals, which are characterized by expanded vocabularies, increased syntactic complexity, simpler sound combinations, more regular and simplified morphologies, greater compositionality, and enhanced semantic transparency (Benítez-Burraco and Kempe, 2018). We should not expect to find direct evidence of the enhancement of such features in the WS language compared to the neurotypical language, because people with WS exhibit moderate-to-severe disabilities in different cognitive domains, including mental retardation, with a noteworthy impact on their language abilities. As noted by Karmiloff-Smith and Mills (2006: 587), in WS one finds “a mixture of delay, deviance, and asynchronies across the developing system.” At the same time, it is true that in some domains, subjects with WS score better than people with other developmental disorders in spite of deep underlying deficits, suggesting that compensatory mechanisms are active (see Karmiloff-Smith, 1998 and Mervis and Becerra, 2007 for discussion). Accordingly, if we want to use WS as a model for the effects of self-domestication in language (evolution), it makes more sense to compare it with other cognitive disorders also entailing abnormal domesticated features and language deficits, particularly, with ASD. Some overlap exists between both conditions. Hence, both ASD and WS are characterized by anxious behaviors and attention deficits (Ng et al., 2018), some of the genes within the WS region are also candidates for ASD (Sanders et al., 2011), and individuals with WS have some risk of suffering from autistic-behaviors (Tordjman et al., 2012; Klein-Tasman et al., 2018). Nonetheless, the ASD phenotype grossly mirrors the WS phenotype. This is particularly true regarding domestication, because features of domestication are attenuated in people with ASD (Benítez-Burraco et al., 2016).

We have good accounts of the WS language (see Karmiloff-Smith and Mills, 2006; Brock, 2007; Mervis and Becerra, 2007; Martens et al., 2008 for good reviews) and the ASD language (see Rapin and Dunn, 2003; Tager-Flusberg and Joseph, 2003; Sterponi et al., 2015 for good reviews), but very few studies comparing the WS and the ASD phenotypes in the communicative domain (see Asada and Itakura, 2012; Lacroix et al., 2016). Overall, although both subjects with ASD and subjects with WS exhibit impaired social cognition and communicative skills (see Asada and Itakura, 2012 for detailed discussion), the pragmatic abilities of autistic people are more impaired (Philofsky et al., 2007). Morphology in children with ASD is usually impaired: they omit certain morphemes (articles, auxiliary forms, past tense), which emerge quite later (Bartolucci et al., 1980; Roberts et al., 2004; Modyanova et al., 2017). They also have problems with complex syntactic structures, like passives (Tager-Flusberg, 1981) or relative clauses (Riches et al., 2010). By contrast, in children with WS, regular morphology is quite preserved (they experience more problems with irregular forms), although they have problems with complex syntax too (particularly, relative clauses) (see Karmiloff-Smith and Mills, 2006; Mervis and Becerra, 2007; Martens et al., 2008). The only direct comparison between the ASD language and the WS language suggests that children with ASD suffer from specific grammar impairments (i.e., the inability to bind a pronoun to its antecedent) that are not observed in children with WS, who perform like typically developing younger children (Perovic et al., 2013). Regarding vocabulary and semantic knowledge, children with WS are typically reported to excel at expressive vocabulary and although they experience problems with providing definitions of words and correct sentence comprehension, they exhibit normal semantic organization and fluency (Volterra et al., 1996; Mervis et al., 1999, Purser et al., 2011; Van Den Heuvel et al., 2016; see Mervis and Becerra, 2007 for discussion). On the contrary, lexical knowledge is delayed in children with ASD (Chawarska et al., 2007; Herlihy et al., 2015). Only for individuals who acquire a good language command, vocabulary is reported to be a relative strength (Kjelgaard and Tager-Flusberg, 2001; Mayes and Calhoun, 2003). Moreover, although children with ASD seemingly rely on the same cognitive and perceptual machinery for lexical development as typically developing children, these learning mechanisms are less efficient (Arunachalam and Luyster, 2016). For example, they are able to rely on gaze cues for inferring word meaning, but they fail in using cues of speaker reference or intention (Jing and Fang, 2014). Likewise, they exhibit problems extending word meanings and situating them in semantic networks, which are organized differently to neurotypical children (see Arunachalam and Luyster, 2016). Interestingly, ASD and WS exhibit opposite brain responses (N400) to tasks involving semantic integration (Fishman et al., 2011).

As noted above, because domestication gives rise to neoteny and enhanced sociability, it has been hypothesized to favor parenting and learning behaviors, as well as input enhancement by parents and other caregivers, which facilitates the acquisition of complex languages (see Benítez-Burraco and Kempe, 2018 for discussion). Interestingly, children at risk or with ASD show reduced preference for infant-directed speech (Watson et al., 2012), this impacting negatively on language skills at later ages (Nadig et al., 2007; Paul et al., 2007; Watson et al., 2010). Evidence about WS children is not available, but compared to children with 22q11.2 Deletion syndrome, who commonly exhibit autistic features (Ousley et al., 2017), they express more positive emotions toward their mothers in conflict interaction, higher levels of child’ engagement, and enhanced reciprocity (Weisman et al., 2015).

Additionally, domestication increases play behavior in animals (Hart, 1985; Himmler et al., 2013; Kaiser et al., 2015). Enhanced play behavior has been hypothesized to contribute to language complexity too (see Langley et al., unpublished for detailed discussion). Several reasons support this view. First, play (particularly, pretend and symbolic play) and language root on similar cognitive and social skills (Weisberg, 2015). Second, they are linked through common ontogenetic roots (Piaget, 1962; Belsky and Most, 1981). Third, they are mutually supportive behaviors (Vygotsky, 1962; Bruner, 1983; Levy and Gottlieb, 1984; Quinn et al., 2018). Finally, play helps the developing child to gain language exposure (and thus access richer and more varied language structures and uses) and language practice (usually in the form of playful child-directed activities like songs and nursery rhymes) (Bebout and Belke, 2017). Although children with WS commonly show problems in their functional play, creativity, and imagination, they exhibit spared abilities for correctly responding to joint attention or for sharing enjoyment and requesting (Klein-Tasman et al., 2007). During social and play interaction the behavior of children with WS is predominantly dyadic, but not triadic (Laing et al., 2002). Compared to typically developing children matched for linguistic abilities, children with WS exhibit less spontaneous functional play and imaginary play, although their abilities for symbolic play correlate with their expressive and receptive language, like in the typically developing population (Papaeliou et al., 2011). By contrast, children with ASD exhibit reduced ability to respond to joint attention (Mundy et al., 1994; Charman et al., 2004), with joint attention disabilities strongly correlating with language impairment (Bono et al., 2004; Bottema-Beutel, 2016), although symbolic and pretend play levels also correlate with language abilities (Hobson et al., 2013).

## Conclusion

Overall, it is difficult to launch any robust conclusion about the effect of the enhanced socialization, potentially resulting from their hyper-domesticated phenotype, on the language abilities and features of children with WS compared with children with cognitive disorders entailing impoverished social function, like ASD. A reason is that although in WS prosocial aspects of social functioning are not usually impaired, difficulties with the social-cognitive aspects of social functioning are frequently observed, impacting negatively on communication and cognition (Klein-Tasman et al., 2011). Nonetheless, considering the evidence discussed in this paper, it seems plausible that enhanced features of domestication might contribute to explaining their linguistic profile, and in particularly, their language strengths compared to other cognitive disorders. All children with disorders need extra input enhancement and scaffolding of language acquisition to improve their language disabilities, but it might be hypothesized that to some extent in WS this is partially provided by their enhanced self-domesticated features. Obviously, this is an empirical question that we expect to address in the near future. Accordingly, we have designed an experiment to compare how artificial grammars are learned and transmitted by children with ASD and with WS (due to the reduced visual abilities and notable hearing abilities of the latter, we have found it is more appropriate to rely on sounds instead of pictures). We hypothesize that the grammars learned by children with WS will acquire some exoteric features as they are transmitted along a chain of learners. Incidentally, the possibility that one important etiological factor of the observed deficits in WS is the hypofunction of the NC (caused by the downregulation of the genes highlighted in section “Genetic Signatures of Domestication and the Genetics of WS) is worth exploring in detail too. All in all, the evidence reviewed in this paper reinforces the view that a deep link exists between (self-)domestication, language evolution, and language impairment, and that it is worth examining this link in detail if we want to gain a more accurate view of how language evolved in our species as a result of changes that are biological and cultural by nature.

## Author Contributions

AB-B conceived the paper, wrote the sections “Introduction,” “Genetic Signatures of Domestication and the Genetics of WS,” “WS, Domestication, and Language Evolution,” and “Conclusion.” AN wrote the section “Domestic Features in WS.” Both authors revised and approved the final manuscript.

## Conflict of Interest Statement

The authors declare that the research was conducted in the absence of any commercial or financial relationships that could be construed as a potential conflict of interest.

## Footnotes

1 http://omim.org/

2 https://decipher.sanger.ac.uk/

3 https://string-db.org/

4 amp.pharm.mssm.edu/Enrichr

5 http://hbatlas.org

## Supplementary Material

TABLE S1 Set of genes considered in this study and predicted functional links between selected genes according to String 10.5.

TABLE S2 GO analyses via Enrichr of candidate genes for domestication that are significantly dysregulated in the blood of patients with WS.

TABLE S3 Brain expression profile according to the Human Brain Transcriptome Database of candidate genes for domestication that are significantly dysregulated in the blood of patients with WS. Six different brain regions are considered: the cerebellar cortex (CBC), the mediodorsal nucleus of the thalamus (MD), the striatum (STR), the amygdala (AMY), the hippocampus (HIP) and 11 areas of neocortex (NCX).

TABLE S4 GO analyses via Enrichr of candidate genes for NC development and function that are significantly dysregulated in the blood of patients with WS.

TABLE S5 Brain expression profile according to the Human Brain Transcriptome Database of candidate genes for NC development and function that are significantly dysregulated in the blood of patients with WS. Six different brain regions are considered: the cerebellar cortex (CBC), the mediodorsal nucleus of the thalamus (MD), the striatum (STR), the amygdala (AMY), the hippocampus (HIP) and 11 areas of neocortex (NCX).
